# Clinical application of cannulated screws tension band and cerclage fixation for displaced patellar fractures based on fracture morphological classification

**DOI:** 10.3389/fsurg.2026.1808022

**Published:** 2026-07-14

**Authors:** Yong-Long Qiu, Ya-Ping Xiao, Ling Li, Jian-Zhong Chang

**Affiliations:** 1 The Department of Orthopedic Surgery, Wuhan Third Hospital, Tongren Hospital of Wuhan University, Wuhan, China; 2 The Department of Orthopedic Surgery, CR & WISCO General Hospital Affiliated to Wuhan University of Science and Technology, Wuhan, China

**Keywords:** cannulated screw, cerclage fixation, internal fixation, patellar fractures, tension band

## Abstract

**Introduction:**

Numerous surgical strategies are available for displaced patellar fractures, yet a universally accepted treatment standard remains lacking. Internal fixation constructed on the tension-band principle constitutes the primary treatment modality for such injuries. This study aimed to evaluate the clinical outcomes of two cannulated screw tension-band fixation techniques combined with cerclage fixation for displaced patellar fractures.

**Methods:**

A retrospective study was conducted on patients with displaced patellar fractures. The patients were divided into the group I treated with modified cross cannulated screws tension band and cerclage fixation, and the group II treated with parallel cannulated screws tension band and cerclage fixation. Baseline data, intraoperative variables, fracture healing status, complications, and Böstman scores were compared between the two groups to assess clinical efficacy.

**Results:**

All patients in both groups were followed up for 2 years. Two patients in Group II presented with anterior patellar discomfort, whereas no adverse events were recorded in Group I. Bony union was achieved in all cases of both groups. There were no significant differences in incision length, hospital stay, fracture healing time, or complications between the two groups (*P* > 0.05). The operation time, intraoperative blood loss, and fluoroscopy times in the group I were higher than those in the group II (*P* < 0.05). The Böstman scores of both groups at 6 months after surgery were significantly lower than those before surgery (*P* < 0.05), and there was no statistically difference between the two groups at 6 months after surgery (*P* > 0.05).

**Conclusions:**

Both cannulated screws tension band combined with cerclage fixation can achieve good clinical efficacy in the treatment of patellar fractures. The operation time, intraoperative blood loss, and fluoroscopy times in the cross cannulated screws tension band group are higher than those in the parallel cannulated screws tension band group. In clinical practice, the cross screw insertion method can be considered when fracture comminution makes parallel screw placement difficult.

## 1 Introduction

Patellar fracture is a common type of fracture in clinical traumatology, with an incidence accounting for approximately 10% of all fractures (1). Since the patella can increase the strength of the quadriceps by about 30% through the lever action during knee extension, fractures mainly significantly affect knee extension movement (2, 3). No matter which surgical approach is utilized, given the patella's central role in the patellofemoral joint, surgeons must restore a smooth articular surface after patellar fracture repair to lower the incidence of traumatic patellofemoral arthritis and prevent complications such as knee flexion contracture. Inadequate intraoperative management may trigger multiple adverse sequelae, including fracture nonunion, intra-articular adhesion, post-traumatic arthritis, loss of fixation, and symptomatic hardware (4).

Operative fixation is routinely recommended for patellar fractures with an articular surface displacement greater than 2 mm (5). Currently, the commonly used treatment methods mainly include Kirschner wire tension band, cannulated screw tension band, shape memory alloy patellar concentrator, circumferential suture cerclage of the patella, absorbable material fixation, and patellar ring (1, 6, 7). All the aforementioned fixation techniques possess distinct merits and limitations for patellar fracture management, and a unified gold-standard surgical protocol has not yet been established (1, 7). At present, most treatment methods adopt the tension band principle to ensure continuous dynamic compression of the patella during movement, avoid fracture displacement, and promote fracture healing. Kirschner wire tension band fixation is the most widely used clinical treatment method (8, 9). Tensile loads on the patella during knee motion transform into compressive force at the fracture ends, permitting early functional exercise and reducing immobilization complications such as knee stiffness. Nevertheless, internal fixation-associated complications are reported in 22% to 53% of patients, including Kirschner wire loosening, cutaneous irritation, and surgical site infection (10–12). Given the smooth outer surface of Kirschner wires, these implants fail to secure bone fragments and are unable to generate axial compressive stress across the fracture site (13). Cannulated screws achieve superior bone purchase compared with Kirschner wires, which correspondingly lowers the risk of implant loosening (14, 15). The screw tail can be buried deep within bone tissue, resulting in minimal soft tissue irritation. Cannulated screws deliver compressive force to fracture fragments intraoperatively and preserve consistent compressive stress at the fracture site during postoperative knee extension. Cannulated screw tension band is superior to Kirschner wire tension band in terms of excellent and good rate of fracture reduction and postoperative recovery of knee joint function (12, 14–16).

Given the above mechanical and clinical advantages, cannulated screw tension-band fixation is the primary operative modality utilized for displaced patellar fractures at our institution. Accordingly, we performed a retrospective comparative study to investigate this fixation technique. This retrospective study enrolled patients with displaced patellar fractures admitted between February 2021 and February 2023. Subjects received either modified crossed cannulated screw tension-band fixation or parallel cannulated screw tension-band combined with cerclage fixation. The clinical efficacy of these two internal fixation methods in the treatment of patellar fractures was compared to aim to provide a reference for the clinical selection of appropriate surgical methods for patellar fractures. Clinically, simple transverse patellar fractures and comminuted complex patellar fractures differ greatly in biomechanical stability and internal fixation requirements. It is inappropriate to directly compare cross and parallel cannulated screw fixation as equivalent alternatives for the same fracture pattern. Parallel screw configuration is biomechanically suitable for routine transverse fractures, while cross screw placement is mostly reserved for severely comminuted fractures with insufficient bone bed for parallel screw insertion. Therefore, this study aimed to observe the clinical effect of two screw placement strategies based on different fracture characteristics, rather than a simple head-to-head superiority comparison.

## 2 Materials and methods

### 2.1 Study design and selection criteria

**Figure 1:**
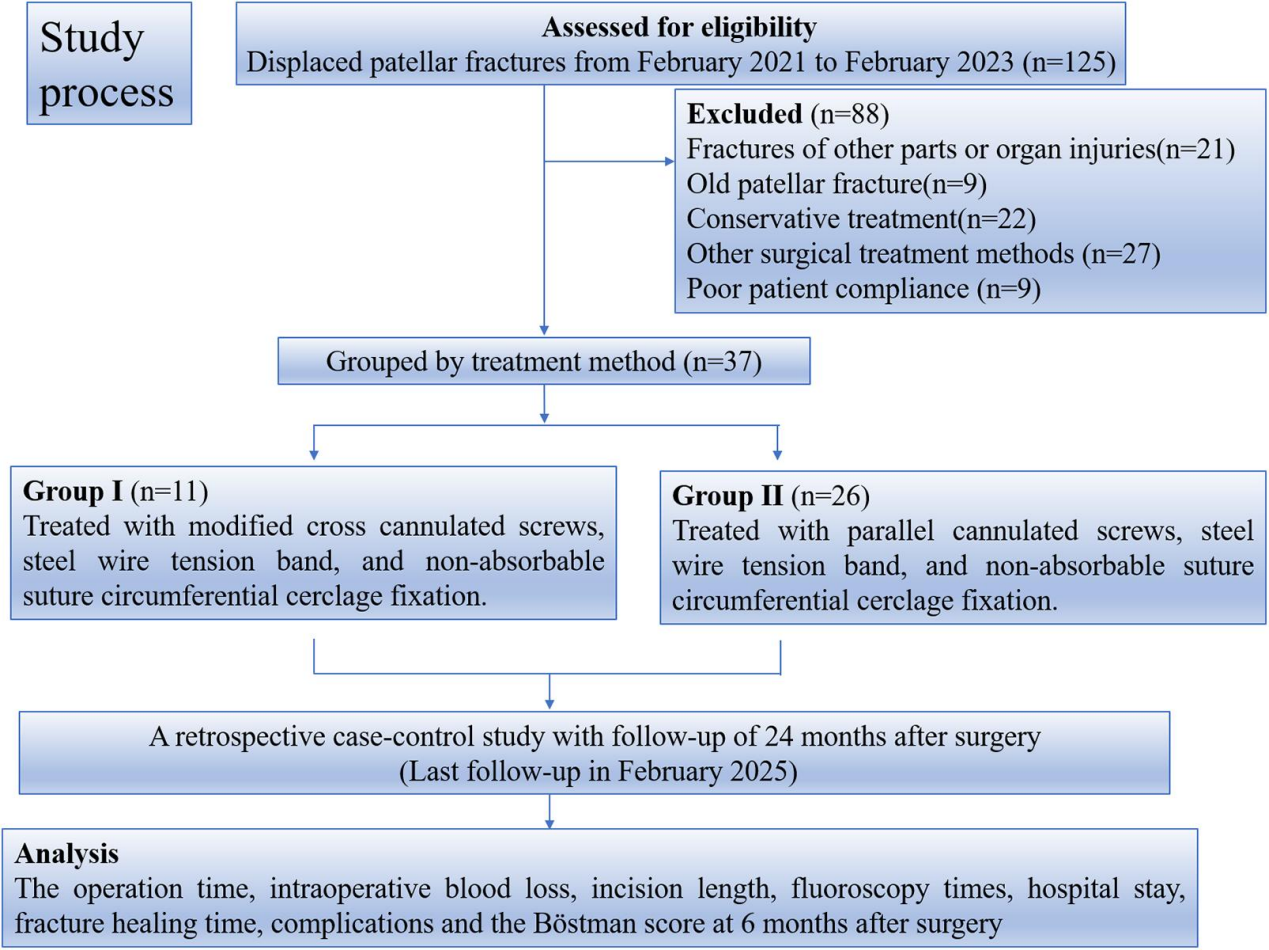
The flow chart of the study.

This study is a retrospective study. The detailed process of the research can be found in Figure 1. Inclusion criteria were as follows: 1) Fresh patellar fracture with a disease duration of less than 3 weeks; 2) Displaced patellar fracture classified by the Rockwood classification, treated with cannulated screw tension-band fixation; 3) Closed fracture without open soft tissue injury; 4) Absence of severe underlying comorbidities that compromise surgical outcomes or constitute surgical contraindications; 5) Satisfactory patient compliance to complete standardized postoperative rehabilitation and regular follow-up.

Exclusion criteria were as follows: 1) Concurrent fractures at other anatomical sites or concomitant visceral injuries;; 2) Old patellar fractures (duration > 3 weeks); 3) Patients managed with conservative therapy, either due to voluntary refusal of surgery or existing surgical contraindications; 4) Receipt of alternative surgical interventions, including patellectomy, K-wire tension-band fixation, plate internal fixation, etc.; 5) Inadequate patient compliance during follow-up and rehabilitation.

All enrolled patients provided written informed consent for surgical procedures and implant implantation. This clinical trial was reviewed and approved by the Institutional Ethics Committee of our hospital.

### 2.2 Population and setting

A total of 37 patients with displaced patellar fractures admitted between February 2021 and February 2023 were enrolled in the present study and stratified into Group I and Group II based on the distinct surgical fixation techniques they received. Group I (11 patients) underwent fixation using modified crossed cannulated screws (titanium alloy, 3.5 mm diameter; Tianjin Zhengtian Medical Devices Co., Ltd., Tianjin, China), 1 mm-diameter stainless steel wire tension bands, and circumferential cerclage with 1-0 non-absorbable sutures (Beijing Deyidamei Medical Technology Co., Ltd., Beijing, China) (Figure 1). Group II (26 patients) received fixation with parallel cannulated screws (titanium alloy, 3.5 mm diameter; Tianjin Zhengtian Medical Devices Co., Ltd., Tianjin, China), 1 mm stainless steel wire tension bands, and circumferential cerclage using 1-0 non-absorbable sutures (Beijing Deyidamei Medical Technology Co., Ltd., Beijing, China) (Figure 1). All patients received preoperative anteroposterior and lateral knee radiographs as well as knee computed tomography (CT).

### 2.3 Surgical techniques

**Figure 2:**
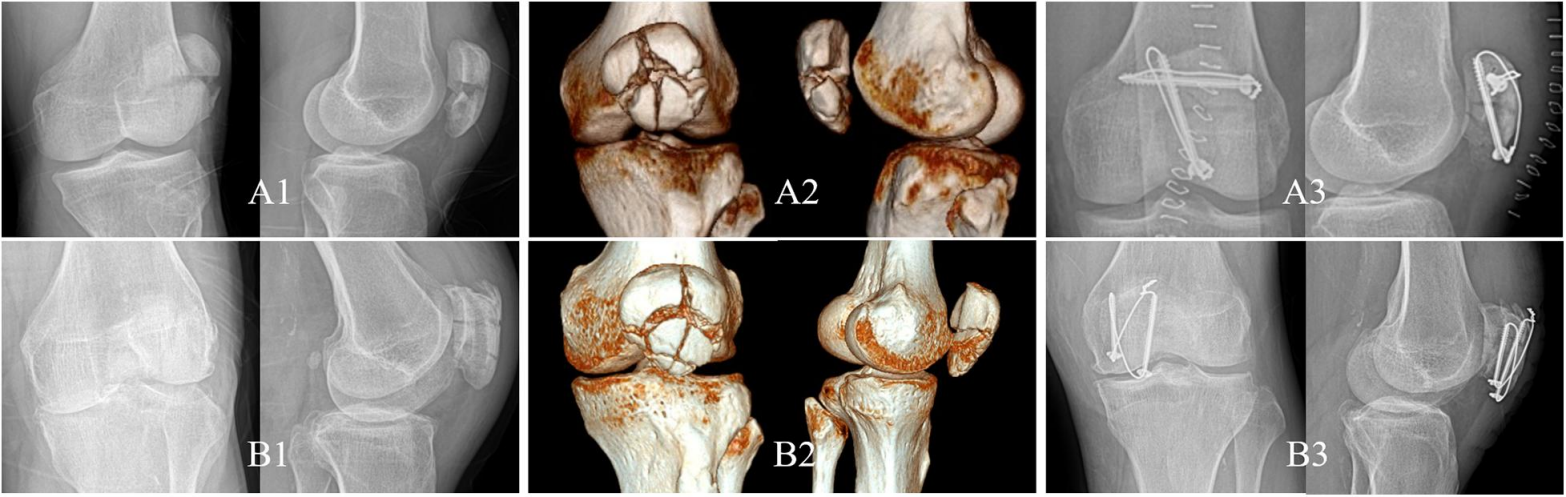
Illustrations of displaced patellar fractures treated with two cannulated screw tension-band fixation techniques augmented by 1-0 non-absorbable suture cerclage: modified crossed cannulated screw tension-band fixation versus parallel cannulated screw tension-band fixation. **(A)** A 54-year-old male patient presenting with comminuted patellar fracture managed with modified crossed cannulated screw tension-band fixation combined with 1-0 non-absorbable suture cerclage: **A1** Preoperative anteroposterior and lateral radiographs; **A2** Preoperative three-dimensional reconstructed computed tomography images; **A3** Anteroposterior and lateral radiographs obtained at postoperative day 2. **(B)** A 68-year-old female patient with comminuted patellar fracture receiving parallel cannulated screw tension-band fixation combined with 1-0 non-absorbable suture cerclage: **B1** Preoperative anteroposterior and lateral radiographs; **B2** Preoperative three-dimensional reconstructed computed tomography images; **B3** Anteroposterior and lateral radiographs acquired 2 days postoperatively.

Fracture classification according to Rockwood classification is carried out to guide the planning of preoperative surgical procedures (17). All patients in both groups were given spinal anesthesia and placed in the supine position. Routine sterile preparation and draping were performed on the affected knee, followed by inflation of a pneumatic tourniquet. A midline longitudinal incision was created anterior to the patella to expose the superior and inferior patellar poles, fracture site, and patellar tendon. The joint cavity was thoroughly irrigated to evacuate hemarthrosis, interposed soft tissue, and hematoma, followed to identify concomitant ligamentous injuries. Fracture fragments were anatomically reduced under direct visualization to restore congruity of the articular surface, then temporarily held with reduction forceps to preserve the articular step-off alignment. Intraoperative C-arm fluoroscopy was used to confirm adequate fracture reduction. For Group I patients with more complex fracture configurations, two crossed Kirschner wires were placed obliquely. Intraoperative fluoroscopy was then used to verify appropriate wire positioning. Cannulated drilling was conducted along the Kirschner wires, then two cannulated screws with lengths matching the oblique span of the patella were advanced into the bone and tightened to deliver appropriate compression at the fracture site (Figure 2A). In Group II, two Kirschner wires were placed parallel to the patella and perpendicular to the fracture line. Once fluoroscopy confirmed satisfactory placement of the Kirschner wires, cannulated drilling was carried out over the wires. Two cannulated screws with lengths corresponding to the longitudinal dimension of the patella were inserted through the Kirschner wires and secured within the patella, providing parallel longitudinal compression at the fracture site (Figure 2B). Once screw tails were confirmed fully buried within bone tissue, the Kirschner wires were removed in both groups. Stainless steel wire (1 mm in diameter) was passed through the cannulated screws and wrapped around the anterior patella to establish tension-band fixation. Repeat intraoperative C-arm fluoroscopy was obtained to verify congruity of the patellofemoral articular surface and satisfactory positioning of all implants. Intraoperative passive range of motion of the knee was performed to validate stable internal fixation. Similar to the equatorial cerclage, a circular suture is applied around the periphery of the patella for fixation, which has been previously referred to as cerclage fixation. The surgical wound was irrigated and closed.

### 2.4 Postoperative management

All patients in both groups were routinely given antibiotics to prevent infection before surgery. After surgery, the affected limb was elevated to facilitate detumescence. For elderly patients, low-molecular-weight heparin was given to prevent deep venous thrombosis of the lower extremities according to the patient's thrombus risk. On postoperative day 1, patients initiated isometric muscle contraction and relaxation training with the affected knee maintained in full extension. From 2 to 3 days after surgery, patients performed isometric contraction of the quadriceps and passive flexion and extension exercises of the knee joint, with the range of motion limited to <30°. Once anesthesia subsided, patients were instructed to ambulate with crutches under non-weight-bearing status. Two weeks after surgery, the knee joint was gradually moved to 90°. At approximately 3 to 4 weeks postoperatively, patients were permitted active knee range of motion from 0° to 120°, alongside progressive partial weight-bearing ambulation with crutches. From 4 to 6 weeks after surgery, full-range functional exercises of the knee joint were performed. At 8 weeks postoperatively, patients were allowed full weight-bearing mobilization using crutches and then progressed gradually to normal walking without supports. After discharge, patients were advised to return to the hospital for regular follow-up generally 1 month, 2 months, 3 months, 6 months, 1 year, and 2 years after surgery to guide functional exercises. At approximately 1.5 years postoperatively, implant removal was performed at the patient's discretion.

### 2.5 Data collection

We compared the two groups in terms of operation time, intraoperative blood loss, incision length, fluoroscopy frequency, length of hospital stay, fracture healing time, and postoperative complications.

Fracture healing time was defined as absence of tenderness and axial percussion pain over the patellar fracture site, satisfactory functional range of motion of the affected limb, and radiographic evidence of blurred or obliterated fracture lines.

Complications included internal fixation loosening or migration, loss of reduction, subcutaneous soft tissue irritation, superficial surgical site infection, intra-articular infection, and delayed fracture healing. The knee joint function of the affected limb was evaluated using the Böstman scoring standard at 6 months after surgery (18).

### 2.6 Statistical analysis

SPSS 19.0 statistical software was used for data processing. Measurement data were expressed as mean ± standard deviation (x¯ ± SD). After testing the normality of data with the Kolmogorov–Smirnov test and the homogeneity of variance with the Levene test, independent sample t-test was used for comparison between the two groups if the data were normally distributed and the variances were homogeneous. If the data were not normally distributed or the variances were not homogeneous, Kolmogorov–Smirnov *Z* test or Mann–Whitney U test was used for comparison between the two groups. Count data were expressed as absolute number (%), and chi-square test and Fisher's exact test were used for comparison between groups. *P* < 0.05 was considered statistically significant.

## 3 Results

The Rockwood classification of patients in the group I was mainly type V, while that in the group II was mainly type II and V (Table 1). There were no statistically differences in gender, age, cause of injury, Rockwood classification, fracture type, time from injury to surgery, or preoperative Böstman score between the two groups (Table 1).

**Table 1:** Comparison of baseline data between the Two groups.

Data	Group I	Group II	t/*χ*^2^	*P*
Cases	11	26	-	-
Age (years)	61.3 ± 12.6	60.3 ± 12.5	1.671	0.095
Gender			0.031	0.860
Male (cases)	6 (54.5%)	15 (57.7%)		
Female (cases)	5 (45.5%)	11 (42.3%)		
Cause of injury			0.010	0.919
Traffic injury (cases)	4 (36.4%)	9 (34.6%)		
Fall down (cases)	7 (63.6%)	17 (65.4%)		
Rockwood classification			5.261	0.134
II (cases)	1 (9.1%)	11 (42.3%)	-	-
Ⅳ (cases)	3 (27.3%)	4 (15.4%)		
V (cases)	7 (63.6%)	9 (34.6%)		
VI (cases)	0 (0%)	2 (7.7%)		
Type of fracture				
Closed fracture (cases)	11 (100%)	26 (100%)		
Open fracture (cases)	0 (0%)	0 (0%)		
Time from injury to surgery (days)	2.7 ± 1.2	24 ± 1.2	0.831	0.409
Preoperative Böstman score	17.8 ± 1.6	18.0 ± 1.7	−1.386	0.166

All patients in both groups were followed up for 2 years after surgery. Anterior patellar discomfort was reported in two patients from Group II. By contrast, neither group developed infection, thrombosis, implant loosening or rupture, or symptomatic knee discomfort. Bony union was achieved in all cases of both groups (Table 2). There were no significant differences in incision length, hospital stay, or fracture healing time between the two groups (*P* > 0.05, Table 2). The operation time, intraoperative blood loss, and fluoroscopy times in the group I were higher than those in the group II (*P* < 0.05, Table 2). Regarding the recovery of knee joint function, the Böstman scores of both groups at 6 months after surgery were significantly lower than those before surgery (*P* < 0.05), and there was no statistically difference in Böstman scores at 6 months after surgery between the two groups (*P* > 0.05, Table 2).

**Table 2:** Comparison of clinical data between the Two groups.

Data	Group I	Group II	t/Z/χ^2^	*P*
Operation time (hours)	62.1 ± 4.4	60.7 ± 7.0	5.992	0.000
Blood loss (mL)	85.4 ± 16.5	81.1 ± 14.1	6.163	0.000
Incision length (cm)	10.0 ± 0.75	10.0 ± 0.90	0.920	0.358
Fluoroscopy times (times)	7.1 ± 1.5	6.2 ± 1.8	11.908	0.000
Hospital stay (days)	9.4 ± 4.4	9.2 ± 4.6	0.954	0.340
Fracture healing time (weeks)	12.6 ± 1.1	12.4 ± 1.2	−1.664	0.096
Complications (cases)			0.023	0.880
Anterior patellar discomfort	2（7.7%）	0 (0%)	-	-
None	24（92.3）	11 (100%)	-	-
Böstman scores 6 months after surgery	28.6 ± 0.89	28.9 ± 0.88	−1.126	0.158

## 4 Discussion

Patellar fractures represent a prevalent intra-articular injury in clinical practice. Most cases are complicated by disruption of the extensor apparatus, leading to fracture fragment displacement, malalignment, and incongruity of the patellofemoral articular surface (7, 19). The core therapeutic goals for patellar fractures are to preserve the native patellar anatomy, reconstruct congruent articular surfaces, re-establish continuity of the extensor apparatus, sustain knee joint stability, and fully restore physiological knee flexion and extension function (16, 19). With rigid internal fixation in place, early mobilization can be initiated to reduce the risks of knee stiffness, post-traumatic arthritis, and permanent functional impairment. With the continuous update and development of the concept and application materials of fracture internal fixation in clinical practice, internal fixation has gradually become the mainstream method for the treatment of displaced patellar fractures (4).

Multiple internal fixation techniques are available for patellar fractures. According to the biomechanical characteristics of the patella, the application of tension band internal fixation is the most widely used surgical method (4, 16). As the knee flexes and extends, the patella forms a fulcrum against the femoral condyles, producing distracting tensile stress anteriorly. Patellar tension-band fixation counteracts distracting forces between fracture fragments during knee flexion and extension, driving fragment centripetal convergence to sustain compressive stability at the fracture site. The tension-band technique was devised in accordance with the kinematic properties of the patellofemoral joint. At present, tension-band fixation remains the primary surgical modality for managing patellar fractures. However, there are many types of tension band internal fixation for patellar fractures. Kirschner wire tension band and cannulated screw tension band are currently the most commonly used and widely applied tension band techniques in clinical practice (13).

Kirschner wire tension band internal fixation is the most commonly used traditional surgical method for the treatment of patellar fractures, with relatively reliable biomechanical fixation effect (8, 13). Due to the characteristics of relatively simple operation, reliable efficacy, and low material cost, Kirschner wire tension band internal fixation is still widely used in clinical practice. However, Kirschner wires have certain limitations. Conventional Kirschner wire and stainless steel wire constructs exhibit insufficient mechanical toughness and strength, predisposing patients to complications including wire slippage or breakage and Kirschner wire loosening. Such adverse events compromise fracture stability, induce loss of reduction and articular step-off, and ultimately result in unsatisfactory postoperative knee functional recovery following fixation failure. Given that Kirschner wires are placed through the anterior patellar surface, surgeons face difficulties fully tensioning the cerclage wire. Over-tightening may lead to Kirschner wire protrusion, while residual protruding wire tails can trigger soft-tissue irritation from the implant hardware (1, 10). Especially during knee flexion and extension, the protruding part of the Kirschner wire is likely to irritate the anterior knee soft tissues, causing pain, or forming bursitis, and the foreign body sensation and aggravated pain symptoms under the skin. For complex comminuted patellar fractures, smooth Kirschner wires lack threaded engagement and fail to generate compressive fixation across bone fragments. This limitation frequently results in wire loosening, migration, and loss of cerclage wire stability, triggering secondary fragment displacement and eventual implant failure (11, 12).

The traditional steel wire cerclage fixation technique for patellar fractures can reset each fracture fragment of the patella to converge to the center, but it cannot resist the separation force generated during quadriceps contraction or knee flexion, thus prone to re-displacement of the fracture (20). Due to the instability of steel wire cerclage internal fixation, most patients need auxiliary external fixation after surgery and cannot perform early functional exercises of the knee joint, resulting in unstable efficacy (21). Nevertheless, supplementary cerclage fixation reinforces tension-band constructs, rendering this technique applicable to most patellar fracture patterns and associated with generally favorable clinical outcomes.

Combining the advantages of tension band technique and steel wire cerclage internal fixation, this study used cannulated screw tension band combined with 1-0 non-absorbable suture cerclage fixation for the treatment of displaced patellar fractures. The use of cannulated screw tension band internal fixation for the treatment of patellar fractures retains the advantages of tension band surgery. Threading cerclage wire through the cannulated screw channels not only averts hardware protrusion of both wire and implant but also integrates the cannulated screws, cerclage wire and patella into a unified stable construct. This design eliminates soft-tissue irritation complications stemming from exposed Kirschner wire tails. The intersection angle between the various internal fixation devices after the internal fixation system forms a framework for fixation effectively prevent screw rotation and protruding part. During surgical operation, after the two cannulated screw is inserted into the patellar bone, the fracture end is compressed by the thread, which can realize the compression and anti-rotation of the fracture end, provide sufficient force to maintain the stability of the fracture and sufficient pressure to promote fracture healing. In addition, cannulated screws possess a larger diameter than Kirschner wires and can sustain sufficient holding strength to counteract the dynamic tensile loads produced during knee flexion and extension rehabilitation. This design prevents excessive stress concentration on the cerclage wire, thereby reducing the risk of wire rupture. Finally, circumferential peripatellar cerclage with 1-0 non-absorbable sutures helps reset fractured fragments and reduces the risk of fragment separation. Subgroup analysis of tension band wiring constructs showed that auxiliary suture cerclage was closely related to higher reoperation rate, nonunion rate and fixation failure rate, and suture cerclage was more commonly used in highly comminuted fractures with ≥4 fragments (22). In the present study, we performed circumferential cerclage using 1-0 non-absorbable sutures as an adjunct to cannulated screw tension-band fixation. All included patients attained osseous union, with no cases of nonunion or implant failure observed. This favorable outcome may be attributable to our rigorous inclusion criteria, whereby we excluded severely multifragmentary fractures that necessitated complex combined fixation techniques. This finding further underscores that individualized selection of cerclage modalities tailored to fracture complexity is critical for achieving satisfactory therapeutic outcomes.

Clinical observations indicate that for patellar fractures, particularly comminuted patterns, limited intact bone stock often prevents placement of two parallel cannulated screws. Under such anatomical constraints, we adopted a crossed screw trajectory and achieved successful surgical fixation in all such cases. In the present study, all patients from Group I and Group II completed a 2-year follow-up. Osseous union was documented in every case across both cohorts, while only two patients in Group II reported anterior patellar discomfort. No statistically significant intergroup differences were observed in incision length, hospital stay, or fracture healing time. With respect to knee functional recovery, the Böstman scores of both groups at postoperative 6 months were markedly higher than preoperative values (*P* < 0.05), whereas the two cohorts exhibited comparable Böstman scores at the 6-month follow-up without significant intergroup discrepancy. These results suggest that both screws tension band combined with 1-0 non-absorbable suture circumferential cerclage fixation around the patella can achieve good clinical efficacy. Given the limited thickness of the patella, crossed screw implantation imposes stringent technical demands intraoperatively. Screw trajectories must avoid breaching the articular surface, which would impair patellofemoral motion, and must not perforate the anterior patellar cortex, as this compromises the screw's bony pullout strength. Therefore, the operation time, intraoperative blood loss, and fluoroscopy times of the modified cross screw group were all greater than those of the parallel screw group, which was consistent with our actual intraoperative operation.

Numerous surgical strategies have been described for displaced patellar fractures, each carrying distinct merits and drawbacks. Moreover, head-to-head comparative studies across these techniques have yielded inconsistent conclusions. Accordingly, substantial clinical variability exists in the surgical management of displaced patellar fractures, with treatment selection heavily influenced by individual surgeon preference. Operative approaches are predominantly determined by each surgeon's prior clinical experience and technical proficiency. Cannulated screw tension-band fixation serves as the primary operative modality at our institution. Our data verify that this technique delivers reliable fixation for patellar fractures, with parallel screw trajectory as the preferred implantation strategy. Nevertheless, for severely comminuted fractures where parallel screw implantation is anatomically constrained by inadequate bone stock or limited operative space, a crossed screw trajectory may be adopted as an alternative. Bai et al. (23) studied four internal fixation methods for transverse patellar fractures and found that compared with Kirschner wire tension band, cannulated screw tension band is beneficial to shorten the fracture healing time, improve the postoperative knee joint function score, and reduce the occurrence of postoperative complications. Other study has also found that compared with Kirschner wires, the use of two cannulated screws for the fixation of closed transverse patella fractures has a faster healing rate, a better range of knee joint motion, and fewer hardware complications (10, 11, 24). However, a previous retrospective study found that both cannulated screws and Kirschner wires have low complication rates and postoperative infections, but the failure rate of cannulated screws is twice that of Kirschner wires, and the incidence of symptomatic implants in patients treated with Kirschner wires is twice that of the former (12). A recent study found that compared with modified Kirschner-wire tension band and cannulated-screw tension band fixation, ring-pin tension band has advantages in terms of symptomatic hardware and premature failure, respectively (25). Patella fractures can be treated with cannulated lag screws and FiberWire® with a high rate of primary union (96%) and a low rate of symptomatic implant removal (8%) (26). The implant removal rate compares favorably with alternative constructs, with an equivalent rate of fracture union. All three methods have sufficient stability for daily activities. Tension band wiring using cannulated screws showed a better failure load compared with tension band wiring using Kirschner wires and tension band wiring using ring pins (27). Compared with anterior tension band wiring with cannulated screws, anterior locking plate fixation for comminuted patellar fractures biomechanically provides better primary stability (28).

In recent years, anatomically contoured variable-angle locking plate system has gradually become an important alternative for the treatment of complex comminuted patellar fractures. Plate osteosynthesis exhibits superior biomechanical performance compared with traditional tension band wiring, especially for open fractures, highly comminuted fractures with ≥4 fragments, and OTA 34C3 severe patellar fractures (29). A recent study confirmed that the novel anatomical contoured patellar plating system can achieve reliable bone union and low reoperation and implant-related complication rates in challenging patellar fracture cases, and standardized postoperative knee extension protection protocol is conducive to reducing arthrofibrosis and fixation failure (30).

However, plate fixation also has obvious clinical limitations. Although it provides excellent primary stability for severe comminuted and open patellar fractures, it is associated with a significantly higher rate of symptomatic implant removal than tension band wiring, simple cannulated screw fixation and tendon advancement procedures; no significant differences were found in reoperation, nonunion, fixation failure and deep infection rate among different fixation constructs (22). For conventional displaced patellar fractures without severe comminution or open injury, cannulated screw tension band still maintains the advantages of less trauma, simpler operation and lower implant removal rate, which is more in line with clinical routine practice. In summary, the clinical treatment of patellar fractures has obvious individualization, and the appropriate treatment method should be selected according to the surgeon's proficiency, fracture type and preoperative planning.

This study has several limitations. In addition to the inherent limitations of retrospective design and small sample size, this study also had obvious fracture selection bias: the group I was dominated by Rockwood type V comminuted fractures, while the group II was mainly type II and V transverse or moderately displaced fractures. The difference of fracture morphology itself affects the operation difficulty and perioperative indexes, which further indicates that the two surgical methods cannot be directly compared in the same fracture type, and only suitable for stratified application according to fracture classification. Future multi-center studies should strictly stratify fractures by OTA/Rockwood classification to further verify the efficacy of different internal fixation strategies. This study constitutes a preliminary clinical investigation. Further multi-center, randomized, controlled double-blind trials are required to corroborate its clinical efficacy.

## 5 Conclusion

Both crossed and parallel cannulated screw tension-band fixation augmented with 1-0 non-absorbable cerclage sutures yield satisfactory bony union and knee functional recovery in displaced patellar fractures. However, the crossed screw group demonstrated inferior perioperative profiles, including longer operative duration, greater intraoperative blood loss, and increased fluoroscopy frequency relative to the parallel screw group. This discrepancy can be attributed to the greater technical difficulty and stricter anatomical positioning demands associated with crossed screw implantation. Clinically, parallel cannulated screw tension-band fixation serves as the primary treatment for simple transverse patellar fractures. For severely comminuted fractures where parallel screw placement is anatomically unfeasible, a modified crossed screw technique is preferentially recommended. This fixation regimen matches the biomechanical properties of the patella and aligns with the individualized management principles for patellar fractures.

## Data Availability

The raw data supporting the conclusions of this article will be made available by the authors, without undue reservation.
